# Supplementary material for: Nano-Spray-Dried Levocetirizine Dihydrochloride with Mucoadhesive Carriers and Cyclodextrins for Nasal Administration
Source: Pharmaceutics. 2023 Jan 18;15(2):317. doi: 10.3390/pharmaceutics15020317 (PMC9966248; doi:10.3390/pharmaceutics15020317)
Supplement: Supplementary file 1 [file pharmaceutics-15-00317-s001.zip › pharmaceutics-2134258-supplementary.pdf]

Supplementary

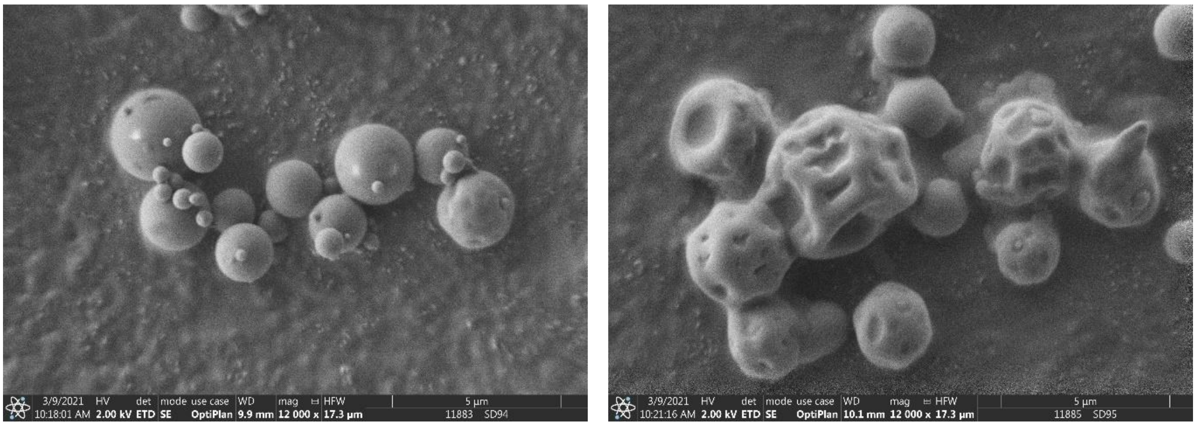

(**a**) (**b**)

Figure S1. SEM images of SD3 (HPBCD) (a) and SD4 (RAMEB) (b) samples.

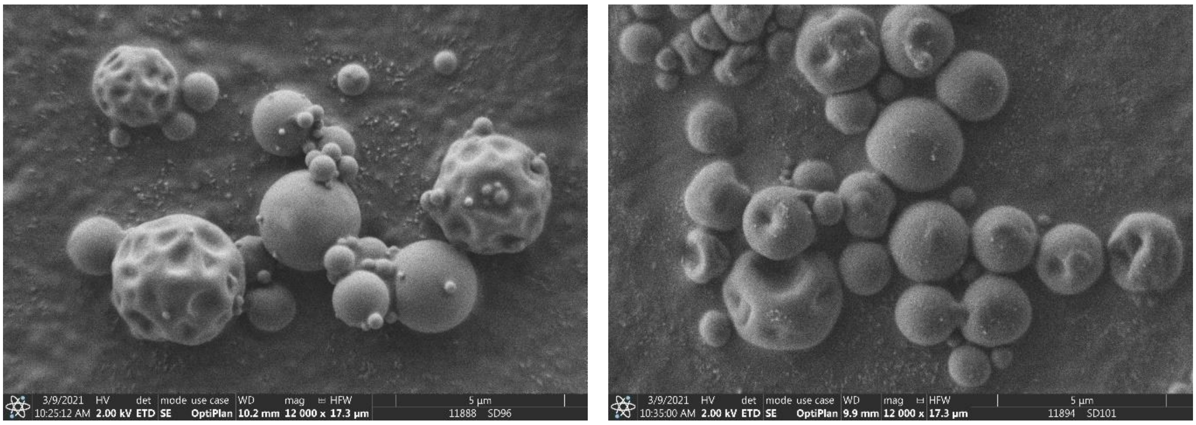

(**a**) (**b**)

Figure S2. SEM images of SD5 (β-CD) (a) and SD8 (HPMC) (b) samples.
